# Dieulafoy on the Treatment of Asthma

**Published:** 1890-03-29

**Authors:** 


### DIEULAFOY ON THE TREATMENT OF ASTHMA.

Dr. Dieulafoy recommends at the commencement of an attack the application by a camel's hair brush to the interior of the nostrils as far up as possible of a five per cent. solution of hydrochlorate of cocaine, or, if preferred, a spray of the same for five minutes at a time. Should this fail to cut short the attack, the patient should inhale five to ten drops of pyrolin. If he be not seen until the attack is at its height, Dr. Dieulafoy would inject 10 grains of the cocaine, repeating it, if necessary, after a quarter of an hour. This we should scarcely advise, since some persons are much more susceptible to its tonic effects than others, though in the inhalation of amyl nitrite we have, it is true, an antidote. When the paroxysm has passed off he relies on iodide of potassium in doses of five to ten grains thrice daily for a fortnight or three weeks, when he substitutes pills of extr. Bellad., and after the principal meal 1·20 grain of arseniate of soda.

